# Clinical Manifestations of Neonatal Hyperbilirubinemia Are Related to Alterations in the Gut Microbiota

**DOI:** 10.3390/children9050764

**Published:** 2022-05-23

**Authors:** Xueli Zhang, Shujuan Zeng, Guoqiang Cheng, Liufang He, Mingqiu Chen, Mingbang Wang, Wenhao Zhou, Huixian Qiu, Zhangxing Wang

**Affiliations:** 1 Division of Neonatology, People’s Hospital of Longhua, Shenzhen 518109, China; zxl22801@163.com (X.Z.); heliufang6022@163.com (L.H.); xiaoqiu1229@hotmail.com (M.C.); 2 Division of Neonatology, Longgang District Central Hospital of Shenzhen, Shenzhen 518116, China; zengshujuansz@163.com; 3 Division of Neonatology, Children’s Hospital of Fudan University, National Center for Children’s Health, Shanghai 201102, China; gqchengcm@163.com; 4 Shanghai Key Laboratory of Birth Defects, Division of Neonatology, Children’s Hospital of Fudan University, National Center for Children’s Health, Shanghai 201102, China; mingbang_wang@fudan.edu.cn (M.W.); zhouwenhao@fudan.edu.cn (W.Z.); 5 Microbiome Therapy Center, South China Hospital, Health Science Center, Shenzhen University, Shenzhen 518116, China

**Keywords:** neonatal hyperbilirubinemia, 16S rRNA gene sequencing, gut microbiota, *Lactobacillus*, *Escherichia coli*

## Abstract

Background and purpose: Neonatal hyperbilirubinemia, also known as neonatal jaundice, is a common and frequent clinical condition with a complex etiology that can lead to brain damage in severe cases. Early recognition of hyperbilirubinemia and timely intervention and treatment can help reduce the occurrence of sequelae. This study was conducted to identify whether the gut microbiota composition can distinguish neonates with hyperbilirubinemia. Methods: Meconium samples were collected from 69 neonates with neonatal jaundice (NJ) and 69 age- and sex-matched neonates without clinically significant jaundice (healthy controls; HCs) for 16S rRNA gene sequencing and microbiome analysis. Results: Compared with HCs, the Chao 1 richness index of the gut microbiota was significantly decreased in the NJ group. The relative abundance of the probiotic gut bacterium, *Lactobacillus*, was significantly lower in the NJ group than in the HC group, whereas the abundances of potentially harmful gut bacteria, such as *Escherichia coli* and *Staphylococcus*, were significantly higher in the NJ group than in HCs. Correlation of the gut microbiota and clinical indicators revealed a positive correlation between *Escherichia coli*/*Staphylococcus* and serum total bilirubin levels. Finally, the results of a random forest machine-learning method to evaluate the possibility of using NJ-associated gut microbiota compositions as potential NJ biomarkers revealed an area under the curve of 96.88%. Conclusions: The abundances of *Escherichia coli* and *Staphylococcus* were positively correlated with serum total bilirubin levels. Hence, the gut microbiota composition is a potential biomarker of NJ.

## 1. Introduction

Neonatal hyperbilirubinemia, also known as neonatal jaundice (NJ), manifests clinically as increased serum total bilirubin levels. Many factors influence human bilirubin metabolism, and persistent or increasing elevated bilirubin is always pathologic [1]. Elevated bilirubin primarily occurs when the bilirubin production rate exceeds the bilirubin excretion rate, thus causing bilirubin to gradually accumulate in the body. Most bilirubin originates from destroyed erythrocytes and is chemically synthesized by the liver to form conjugated bilirubin, which is excreted into the intestines via the gallbladder and bile ducts. In neonates, some of the conjugated bilirubin is reduced to mesobilirubinogen via the gut microbiota. Approximately 80% of the mesobilirubinogen is excreted in the feces, and a small portion is dissociated by the gut microbiota and reabsorbed into the blood through the colon [2]. Studies have shown that the microbial community of newborns begins to colonize during the mother’s pregnancy and that early gut microbiota colonization is critical for formation and maturation of gut microorganisms that affect future growth and immune system development [3]. The gut microbiota is influenced by the mother’s diet and living environment and changes as gestational age increases. After birth, the mode of delivery, feeding, and elements such as prematurity, infection, and asphyxia strongly influence the abundance and diversity of the neonatal microbiota and thus modulate the neonatal immune system response [4]. Our previous study [5] revealed that the gut microbiota plays important roles in the enterohepatic form of bilirubin and bilirubin excretion. In the gut microbiota of patients with NJ, the probiotic bacterium *Lactobacillus lactis* is significantly reduced, and harmful bacteria are significantly increased. These harmful bacteria may be involved in bilirubin metabolism through the galactose metabolic pathway, leading to an imbalance in gut microbiota. This study was conducted to examine the gut microbiota composition of patients with NJ and search for potential gut bacterial markers of NJ.

## 2. Methods

### 2.1. Participants

Participants in this study were patients admitted to the neonatal unit of the People’s Hospital of Longhua District, Shenzhen (Shenzhen, China) from June to September 2018. For full-term infants and for late preterm infants over 35 weeks of gestational age, the diagnostic criteria for NJ were as per the Guidelines for the Diagnosis of Neonatal Hyperbilirubinemia published by the American Academy of Pediatrics [6]. The neonatal hourly bilirubin profile was calculated as per Bhutani et al. and used to diagnose neonatal hyperbilirubinemia when the total serum bilirubin level exceeded the 95th percentile. The inclusion criteria for the NJ group were (1) jaundice within 24 h after birth; (2) total serum bilirubin: >221 μmol/L (12.9 mg/dL) in full-term infants and >257 μmol/L (15 mg/dL) in preterm infants; (3) duration of jaundice > 2 weeks in full-term infants; (4) jaundice receding and reappearing; (5) serum conjugated bilirubin > 34 μmol/L (2 mg/dL); (6) neonates fed orally. If the above guidelines were met, infants were included in the NJ group. Non-jaundiced neonates or those who did not meet the diagnostic criteria for NJ were used as the healthy control (HC) group and were sex- and gestational age-matched with the NJ group. The exclusion criteria were neonates who have previously used antibiotics, jaundice caused by biliary atresia, ultra-premature neonates, sepsis, and congenital genetic metabolic diseases. The Ethics Committee of Shenzhen Longhua District People’s Hospital approved this study (code: 2019ECYJ026, date: 23 February 2019). The participants’ families provided signed informed consent.

### 2.2. 16S rRNA Gene Sequencing

In the neonatal ward, researchers used sterilized disposable stool sampling tubes to collect feces from the newborns within 3 days of birth and avoided contamination from human sources by wearing gloves and masks. Contaminated samples were resampled, and the samples were quickly stored in a −80 °C freezer for later uniform 16S rRNA gene sequencing. The 16S rRNA gene was sequenced as per our previously published articles [7]. Briefly, genomic DNA was extracted using a StoolGen fecal DNA extraction kit (CWBiotech, Beijing, China), and the V4 region of the bacterial 16S rRNA gene was amplified via PCR using 515F and 806R primers. Next, the V4 region of the bacterial 16S rRNA gene was amplified via PCR using a TruSeq^®^ DNA PCR-Free Sample Preparation Kit (Illumina, San Diego, CA, USA) to construct amplicon libraries and sequenced using a HiSeq2500 platform (Illumina). Raw data were merged and filtered for low-quality sequences using QIIME2 (version 2019.10.0) [8]. Uparse software (version 7.0.1001) was used to identify operational taxonomic units (OTUs) with ≥97% similarity. Finally, the SILVA database (version Silva.nr_v128) was referenced for annotation using USEARCH software (version 11.0.98).

### 2.3. Gut Microbiota Analysis

Gut microbiota polymorphisms were analyzed using the QIIME2 diversity plugin and presented as box plots. Principle coordinates analysis (PCoA) was performed using the vegan package in R. For the jaundice-related gut microbiota composition analysis, the taxonomic and functional composition data at the phylum, family, genus, and species levels were used to find the jaundice-related gut microbiota taxonomic and functional compositions using linear discriminant analysis (LDA) effect size (LEfSe). The screening criteria for significantly different gut microbiota species compositions using LEfSe software were α ≤ 0.05 and an LDA score threshold ≥ 2.5.

## 3. Results

### 3.1. Participant Information and Composition

We collected meconium samples from hospitalized neonates during hospitalization; among the participants, 69 were enrolled in the NJ group, and 69 were enrolled as matched HCs. Among them, 55 were boys; 83 were girls; 41 were delivered by cesarean section; 97 were delivered vaginally; 19 were premature, and 119 were full-term. The serum total bilirubin level in the NJ group ranged from 170.5 umol/L to 446.2 umol/L, and the TSB level > 95th percentile for age in hours based on a nomogram for hour-specific serum bilirubin concentration.

### 3.2. Shannon Diversity Indices Were Significantly Lower in the Gut Microbiotas of Neonates with Hyperbilirubinemia

**Figure 1:**
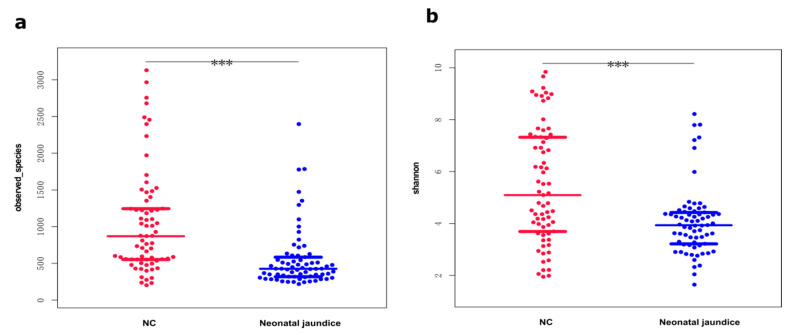
Diversity and variability of the neonatal gut microbiota. The same color is used for the same group of samples; red represents healthy controls; blue represents neonatal jaundice, and each point represents one sample. (**a**) Species richness in the feces of neonates with jaundice and healthy controls; few samples in either group had >2000 microbial species. Most were <1000, and the NJ group had significantly fewer gut microbes. (**b**) Diversity of fecal microorganisms in both groups. The vertical coordinate represents the Shannon index, with larger values indicating a higher response biodiversity (i.e., more species meant that the samples were more evenly distributed). The fecal microbiota diversity was significantly lower in neonates with jaundice than in the controls. *** *p* value < 0.001.

After performing 16S rRNA gene sequencing, we counted the community compositions per sample at the phylum, order, family, genus, and species levels for 69 neonates with hyperbilirubinemia (the NJ group) and 69 matched HCs and then compared the differences between the two groups. The gut microbiota compositions of the NJ group were altered and exhibited a significantly lower gut microbiota richness (<2500 species) compared with that of HCs (>3000 species; Figure 1a). Comparing the gut microbial diversities between the groups revealed significantly lower species diversity and Shannon diversity indices in the NJ group than in HCs (Figure 1b).

### 3.3. Species Compositions of Gut Microbiota Partially Distinguished Neonatal Hyperbilirubinemia

**Figure 2:**
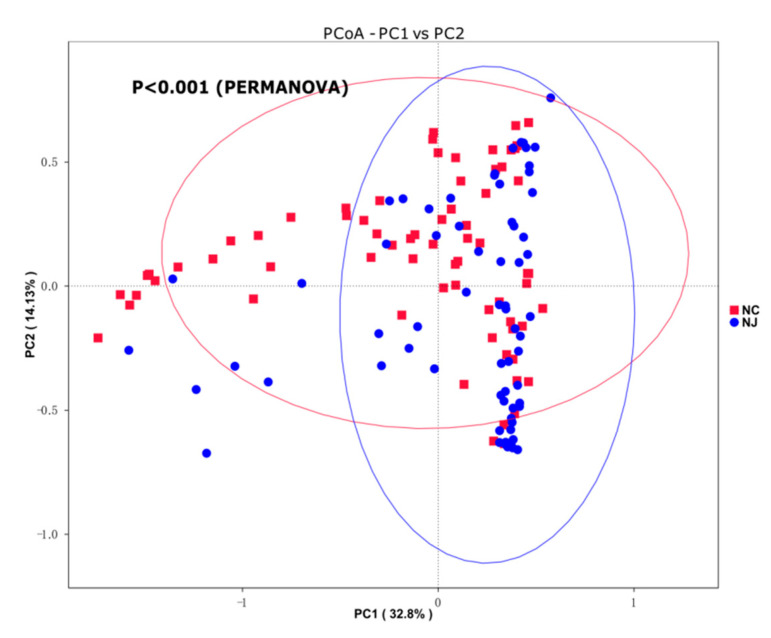
Significant differences in gut microbiotas between neonates with jaundice and healthy controls. The PCoA values based on weighted UniFrac distance analysis differed significantly between the two groups, and the differences in gut microbiota compositions were significantly higher in the control group (PC1; 32.8%) than in the jaundice group (PC2; 14.13%) by nonparametric multivariate ANOVA (*p* < 0.001).

To better understand gut microbiota diversity between groups, we used beta diversity analysis to compare the gut microbiota compositions. First, we performed OTU clustering and species classification analysis on the valid data and PCoA via the multivariate method based on weighted UniFrac distances. Principal component (PC) 1 explained 32.8% of the gut microbiota composition, and PC2 explained 14.13% of the gut microbiota composition. Therefore, the gut microbiota composition can partially distinguish patients with NJ or the HCs (Figure 2). Nonparametric multivariate analysis of variance (PERMANOVA) results showed that the gut microbiota compositions differed significantly between the NJ and HC groups (*p* < 0.001).

### 3.4. Significant Differences in Gut Microbiota Compositions between Groups

**Figure 3:**
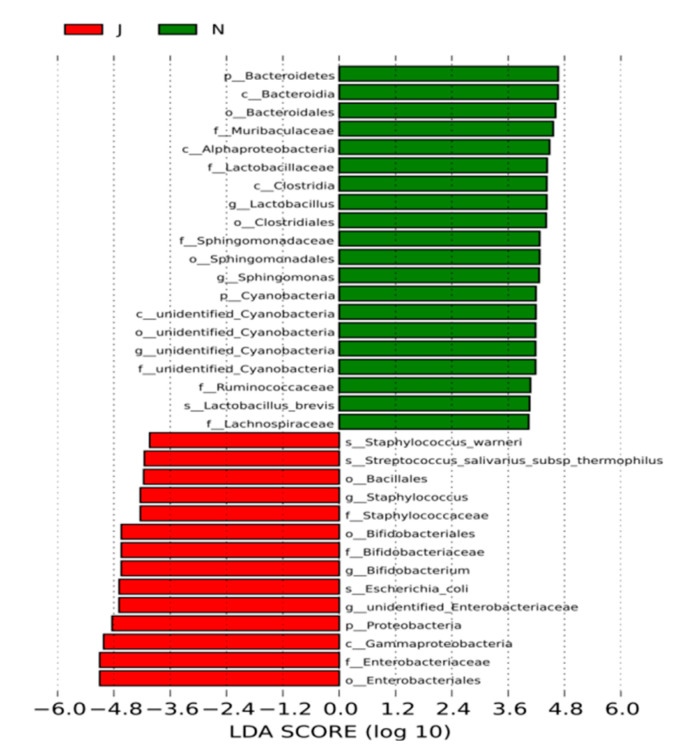
Fecal microbiota compositions differed significantly in neonates with jaundice. Green represents the healthy controls; red represents the jaundice group. p, c, o, f, g, and s represent phylum, class, order, family, genus, and species, respectively. The dominant microbes of the neonatal jaundice group differed significantly from those of healthy controls at all levels.

**Figure 4:**
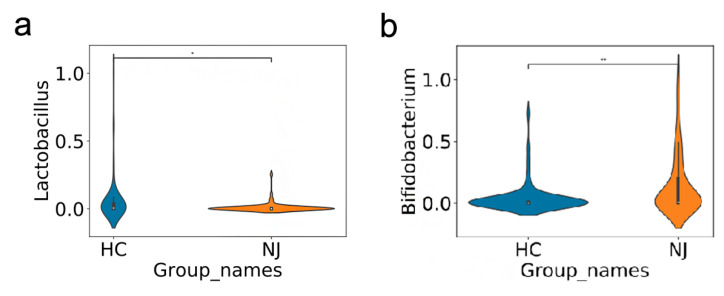
The composition ratios of bifidobacteria and lactobacilli varied between the groups. The same color is used for the same group of samples; blue represents healthy controls; yellow represents neonatal jaundice. (**a**) Bacterial compositions differed in the stool samples of both groups; the vertical coordinate represents the bacterial composition ratio; larger values indicated higher proportions in the group where the organism was located. (**b**) Numbers of species detected in the stools of the neonatal jaundice group and the healthy controls; the bifidobacterial distribution was higher in the neonatal jaundice group; the *Lactobacillus* distribution was significantly lower. *Bifidobacterium* was the dominant microorganism in the feces of jaundiced neonates, and the *Lactobacillus* content was significantly lower than that in the control group. * *p* value < 0.5, ** *p* value < 0.01.

The gut microbiota richness and diversities differed between the NJ group and HCs. To better understand gut microbiota compositions, we used LEfSe to analyze these compositions between the groups and found significant differences at the phylum, order, family, genus, and species levels (Figure 3). The common gut microbes in the HCs in order from most to least abundant were *Bacillus mimicus*, *Bacillus rhamnosus*, *Bacillus deformans*, *Lactobacillus* spp., *Clostridium* spp., *Sphingomonas* spp., and *Cyanobacteria* spp. None of these microbes were common in the feces of the neonates with NJ, except for *Bacillus deformans* (Figure 3). We analyzed the gut microbiotas of the NJ group, and common microbes in order of most to least abundant were *Escherichia coli*, *Aspergillus*, *Bifidobacterium* spp., *Staphylococcus* spp., *Bacillus* spp., and *Streptococcus salivarius* thermophilic subspecies, all of which were uncommon in the HCs except for *Aspergillus*. Among them, *Bifidobacterium* spp. were significantly more numerous in the NJ group than in the HCs. Conversely, the NJ group had significantly fewer lactobacilli than did the HCs (Figure 4). The results showed that fecal specimens differed significantly in both species distribution and abundance between the groups.

### 3.5. Potential Biomarkers for Diagnosing Neonatal Hyperbilirubinemia

**Figure 5:**
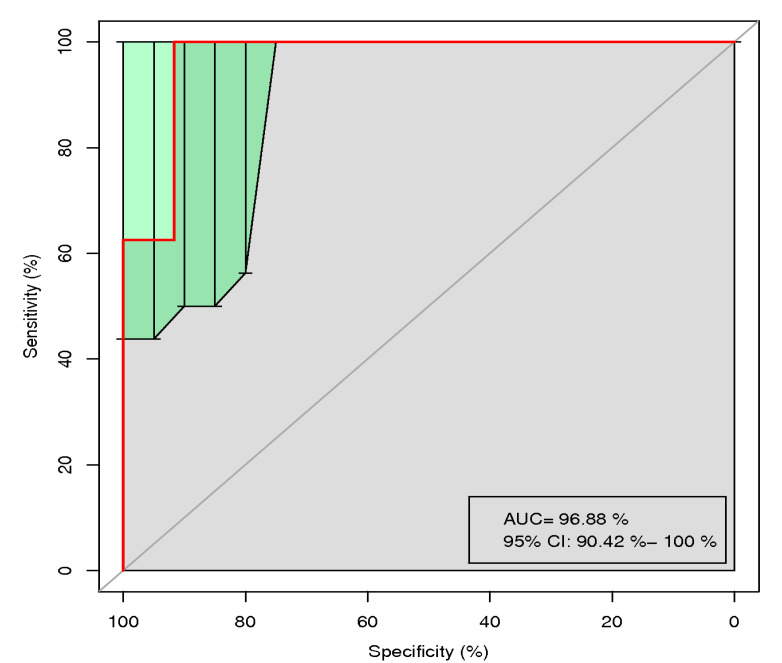
The neonatal gut microbiota composition is a potential biomarker of neonatal jaundice. Random forest analysis of six microbial genera yielded an AUC of 96.88% (95% CI, 90.42–100%), indicating a significant difference between the neonatal jaundice group and the healthy controls.

Fecal microorganisms differed significantly between the NJ group and HCs. To determine whether this difference could be used as a marker for diagnosis of NJ, we performed a random forest analysis using six different *Lactobacillus* spp. from the gut microbiotas of both groups. The results showed an area under the curve (AUC) of 96.88% (95% confidence interval (CI), 90.42%–100%; Figure 5), showing a significant difference between the two groups and thus indicating that the gut compositions of these six *Lactobacillus* spp. may be a potentially useful biomarker for diagnosing NJ.

### 3.6. Gut Microbiota—Bilirubin Association Analysis

**Figure 6:**
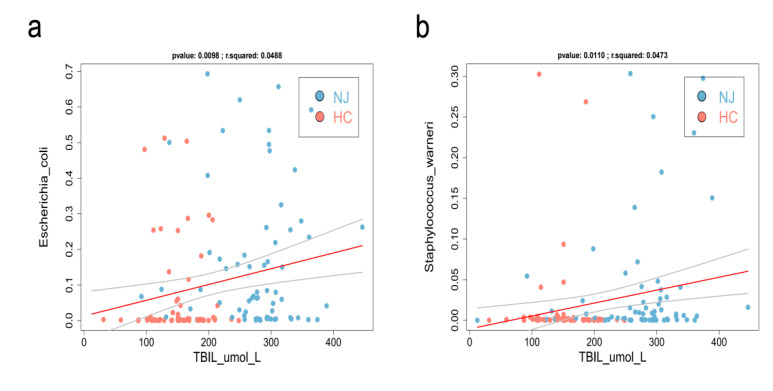
Gut bacteria/clinical indicators association analysis. The gut microbiota affects bilirubin metabolism. We performed a correlation analysis between the gut bacteria and neonatal clinical indicators/bilirubin. The linear relationship between the gut bacteria and clinical indicators was analyzed using the lm function in the regression analysis package of R software (version 4.0). Spearman rank correlation and statistical significance tests were performed using the cor and cor.test functions in R, respectively. Higher neonatal bilirubin values yielded wider distributions of *Staphylococcus* (**b**) and *Escherichia coli* (**a**), and *p* < 0.05 was considered statistically significant.

To further investigate the relationship between gut microbiota and bilirubin, a detectable indicator of neonatal hyperbilirubinemia, we analyzed the gut microbiota-bilirubin association for the gut microbiota and bilirubin values of the NJ group. *Staphylococcus* spp. and *Escherichia coli* were positively correlated with bilirubin levels in the NJ group, indicating that as the numbers of both bacteria increase, they may affect bilirubin metabolism, leading to higher bilirubin levels (Figure 6). Hence, *Staphylococcus* spp. and *Escherichia coli* may be used as effective biomarkers for the diagnosis of NJ.

## 4. Discussion

Jaundice is a relatively common clinical condition in the neonatal period and can be easily treated, but severe pathological jaundice can cause irreversible brain damage in newborns and deserves attention from neonatologists and parents. The gut microbiota plays an important role in bilirubin metabolism in newborns early in life. Our previous studies revealed the involvement of gut microorganisms in bilirubin metabolism. The gastrointestinal tract is the organ with the highest abundance of microorganisms, and studies have shown that the gut microbiota plays an important role in metabolism and immunoregulation and is closely related to human health [9]. More than 350 bacterial species reside in the human intestines [10], including both pathogenic and commensal microorganisms, which interact and influence each other to maintain a dynamic balance. In bilirubin metabolism, the normal gut microbiota of neonates can convert conjugated bilirubin into fecal bilirubinogen, most of which is excreted in the feces. In some neonates, the digestive system is poorly developed, and the gut microbiota is prone to imbalance, which accelerates the bilirubin enterohepatic circulation, leading to neonatal hyperbilirubinemia. Reduced microbial diversity is the main feature of dysbiosis, which can lead to various diseases, such as metabolic disorders [11]. Here, we found that gut species diversity was significantly reduced in neonates with NJ compared with that of HC, which partially affected bilirubin metabolism.

Among the more familiar probiotics in this study, *Lactobacillus* was the dominant microbe in HCs but was relatively deficient in the NJ group, in which it was significantly decreased in abundance and composition ratio, likely owing to its involvement in bilirubin metabolism. *Lactobacillus* spp. have long been recognized as among the most abundant microorganisms in the human gastrointestinal tract and are associated with human gut health [12]. *Lactobacillus* is the major genus of lactic acid bacteria, which improve the gut barrier and play important roles in the human body. Studies have shown that accumulation of higher concentrations of unconjugated bilirubin in the body cause significant cytotoxicity, and treatment with *Lactobacillus plantarum* improves gut oxidative stress, promotes protein kinase activity and expression, and may mitigate the effects of unconjugated bilirubin on apoptosis [13,14]. Some studies have shown that oral *Lactobacillus acidophilus* preparations can help reduce bilirubin levels and shorten the duration of phototherapy treatment and hospital stay [15]. The most effective clinical treatment for neonatal hyperbilirubinemia is blue light irradiation. During consultation and treatment, probiotics containing *Lactobacillus acidophilus* can be administered orally to help children establish a normal gut microbiota network, accelerate gut motility, improve the immune system, reduce hospitalization stay and treatment costs, and improve clinical satisfaction [16].

In this study, 16S rRNA gene sequencing suggested that *Bifidobacterium* was the dominant microorganism in the NJ group. *Bifidobacterium*, as a normal part of the human intestinal microbiota, has immunomodulatory, antitumoral, and anti-inflammatory effects and is closely associated with human health. *Lactobacillus* is considered a beneficial host bacterium with many clinical applications, such as treating diarrhea, balancing gut microbiota, and relieving atopic rashes [17]. *Lactobacillus* supplementation can prevent some gut infections and produce beneficial metabolites to protect the gastrointestinal tract [18]. Some studies [3,4] have confirmed that orally administering *Lactobacillus* preparations to newborns can alter the gut environment, which has positive effects on bilirubin metabolism and reduces the risk of hyperbilirubinemia. Therefore, blue light irradiation combined with probiotics can reduce bilirubin levels and promote bilirubin metabolism in jaundiced children, thus reducing the adverse effects of blue light irradiation to reduce yellowing. Here, we found that changes in the abundance of intestinal probiotics may affect changes in intestinal bilirubin metabolism [16].

Comparing stool samples from the two groups of neonates revealed that *E. coli* was the dominant microbe in the NJ group and was positively correlated with bilirubin. Random forest analysis of *E. coli* showed an AUC of 96.88% (95% CI, 90.42–100%; Figure 4), with a significant difference between the two groups, indicating that *E. coli* is a potentially effective biomarker for diagnosing NJ. *E. coli* is a normal and widely present host bacterium in animal intestines and is an essential clonal host in modern biological studies. However, certain strains of *E. coli* produce enterotoxins under certain conditions and cause infectious diseases [19]. In this study, the NJ group had larger proportions of *E. coli* and *Staphylococcus* than did HCs; *E. coli* and *Staphylococcus* were positively correlated with bilirubin levels and may be involved in the enterohepatic circulation of bilirubin. The exact mechanism of this effect is unclear and requires further study.

Neonatal hyperbilirubinemia is a common and frequent problem in neonates. Unconjugated bilirubin in high concentration can cross the blood–brain barrier and can penetrate the brain cells, which may lead to neuronal dysfunction and death. Severe hyperbilirubinemia can be toxic to the auditory pathways and to the central nervous system, which can result in hearing loss and encephalopathy. If not discovered in time, it will affect the prognosis and quality of life of neonates. The neonatal enterohepatic circulation plays an important role in bilirubin metabolism, and the gut microbiota plays an important role in enterohepatic circulation [5]. In this study, the NJ group showed altered abundances of intestinal probiotic. Furthermore, the abundances of potentially harmful intestinal bacteria were significantly increased, and the abundances of *Staphylococcus* spp. and *Escherichia coli* were positively correlated with serum bilirubin, suggesting that these bacteria are potential markers of neonatal hyperbilirubinemia.

The limitations of this study were that various factors, such as maternal habits, delivery mode, gestational age of the newborn, history of asphyxia, feeding, time of milk initiation, and environmental factors, influence the abundances and composition of the neonatal gut microbiota [4]. Maternal factors are the most influential factors. Additionally, sample size was limited, and we analyzed only the differences in gut microbiotas of two groups of neonates in our hospital. The effects of various influencing factors on the gut microbiota were not further compared or analyzed.

## 5. Conclusions

Changes in the gut microbiota of neonates with hyperbilirubinemia revealed significantly decreased bacterial species abundances. Among them, the abundances of potentially harmful bacteria were increased, while the abundances of beneficial bacteria were decreased. The numbers of *Escherichia coli* and *Staphylococcus* spp. were positively correlated with bilirubin in neonates with NJ. Gut microbiota composition is a potential biomarker of neonatal hyperbilirubinemia. Interventions involving the gut microbiota may provide potential strategies for clinically treating neonatal hyperbilirubinemia.

## References

[1] Lai N.M., Gerard J.P., Ngim C.F., Kamar A.A., Chen K.H. (2021). The Association between Serum Bilirubin and Kernicterus Spectrum Disorder: A Systematic Review and Meta-Analysis. Neonatology.

[2] Lauer B.J., Spector N.D. (2011). Hyperbilirubinemia in the newborn. Pediatr. Rev..

[3] Mutlu M., Irmak E., Aslan Y., Kader Ş. (2018). Effects of *Lactobacillus rhamnosus* GG as a probiotic on neonatal hyperbilirubinemia. Turk. J. Pediatr..

[4] Navarro-Tapia E., Sebastiani G., Sailer S., Toledano L.A., Serra-Delgado M., García-Algar Ó., Andreu-Fernández V. (2020). Probiotic Supplementation during the Perinatal and Infant Period: Effects on Gut Dysbiosis and Disease. Nutrients.

[5] Zhou S., Wang Z., He F., Qiu H., Wang Y., Wang H., Zhou J., Zhou J., Cheng G., Zhou W. (2019). Association of serum bilirubin in newborns affected by jaundice with gut microbiota dysbiosis. J. Nutr. Biochem..

[6] Maisels M.J., Bhutani V.K., Bogen D., Newman T.B., Stark A.R., Watchko J.F. (2009). Hyperbilirubinemia in the newborn infant > or =35 weeks’ gestation: An update with clarifications. Pediatrics.

[7] Xu R., Wu B., Liang J., He F., Gu W., Li K., Luo Y., Chen J., Gao Y., Wu Z. (2020). Altered gut microbiota and mucosal immunity in patients with schizophrenia. Brain Behav. Immun..

[8] Bolyen E., Rideout J.R., Dillon M.R., Bokulich N.A., Abnet C.C., Al-Ghalith G.A., Alexander H., Alm E.J., Arumugam M., Asnicar F. (2019). Reproducible, interactive, scalable and extensible microbiome data science using QIIME2. Nat. Biotechnol..

[9] Butel M.J., Waligora-Dupriet A.J., Wydau-Dematteis S. (2018). The developing gut microbiota and its consequences for health. J. Dev. Orig. Health Dis..

[10] Casaburi G., Duar R.M., Brown H., Mitchell R.D., Kazi S. (2021). Metagenomic insights of the infant microbiome community structure and function across multiple sites in the United States. Sci. Rep..

[11] Weiss G.A., Hennet T. (2017). Mechanisms and consequences of intestinal dysbiosis. Cell. Mol. Life Sci..

[12] Heeney D.D., Gareau M.G., Marco M.L. (2018). Intestinal *Lactobacillus* in health and disease, a driver or just along for the ride?. Curr. Opin. Biotechnol..

[13] Zhou Y., Qin H., Zhang M., Shen T., Chen H., Ma Y., Chu Z., Zhang P., Liu Z. (2010). *Lactobacillus plantarum* inhibits intestinal epithelial barrier dysfunction induced by unconjugated bilirubin. Br. J. Nutr..

[14] Zhou Y.K., Qin H.L., Zhang M., Shen T.Y., Chen H.Q., Ma Y.L., Chu Z.X., Zhang P., Liu Z.H. (2012). Effects of *Lactobacillus plantarum* on gut barrier function in experimental obstructive jaundice. World J. Gastroenterol..

[15] Chen Z., Zhang L., Zeng L., Yang X., Jiang L., Gui G., Zhang Z. (2017). Probiotics Supplementation Therapy for Pathological Neonatal Jaundice: A Systematic Review and Meta-Analysis. Front. Pharmacol..

[16] Deshmukh J., Deshmukh M., Patole S. (2019). Probiotics for the management of neonatal hyperbilirubinemia: A systematic review of randomized controlled trials. J. Matern. Fetal Neonatal Med..

[17] Sanchez B., Ruiz L., de los Reyes-Gavilan C.G., Margolles A. (2008). Proteomics of stress response in *Bifidobacterium*. Front. Biosci..

[18] Yang B., Chen Y., Stanton C., Ross R.P., Lee Y.K., Zhao J., Zhang H., Chen W. (2019). Bifidobacterium and *Lactobacillus* Composition at Species Level and Gut Microbiota Diversity in Infants before 6 Weeks. Int. J. Mol. Sci..

[19] Kaper J.B. (2005). Pathogenic *Escherichia coli*. Int. J. Med. Microbiol..

